# The THERMOSENSITIVE MALE STERILE 1 Interacts with the BiPs via DnaJ Domain and Stimulates Their ATPase Enzyme Activities in *Arabidopsis*

**DOI:** 10.1371/journal.pone.0132500

**Published:** 2015-07-17

**Authors:** Zhao-Xia Ma, Ya-Jun Leng, Guang-Xia Chen, Peng-Min Zhou, De Ye, Li-Qun Chen

**Affiliations:** 1 State Key Laboratory of Plant Physiology and Biochemistry, College of Biological Sciences, China Agricultural University, Beijing, China; 2 National Center for Plant Gene Research (Beijing), Beijing, China; Wuhan University, CHINA

## Abstract

The *Arabidopsis TMS1* encodes a heat shock protein identical to the Hsp40 protein AtERdj3A and plays important roles in the thermotolerance of pollen tubes and other plant tissues. Despite its importance to plant growth and reproduction, little has been known about its mechanisms underlying thermotolerance of plants. In this study, the relationship between TMS1 and the Hsp70 proteins, Binding Immunoglobulin Proteins (BiPs) was explored to understand the molecular mechanisms of *TMS1* in thermotolerance of plants. The expression of *TMS1* was induced not only by heat shock, but also by dithiothreitol (DTT) and L-azetidine-2-carboxylic acid (AZC), similarly to the three *BiP* genes, indicating that *TMS1* may be involved in unfolded protein response (UPR). The firefly luciferase complementary imaging (LCI), GST pull-down and ATPase enzyme activity assays demonstrated that the DnaJ domain of TMS1 could interact with BiP1 and BiP3, and could stimulate their ATPase enzyme activities. In addition, the expression level of *TMS1* was reduced in the *bzip28 bzip60* double mutant. These results suggest that TMS1 may function at the downstream of *bZIP28* and *bZIP60* and be involved in termotolerance of plants, possibly by participating in refolding or degradation of unfolded and misfolded proteins through interaction with the BiPs.

## Introduction

In flowering plants, the male gametes are delivered into the female gametophyte for double fertilization by the pollen tubes. Therefore, growth of pollen tubes is important for successful fertilization. Studies have shown that growth of pollen tubes is sensitive to high-temperature stress [1–6]. In most flowering plants, however, pollination and fertilization occur during hot summers. Thus, the plants must have evolved a mechanism of thermotolerance to maintain normal growth of their pollen tubes at high temperatures [7]. The previous study showed that *THERMOSENSITIVE MALE STERILE 1* (*TMS1*) is required for thermotolerance in *Arabidopsis* pollen tubes and responsive to the heat shock treatment in seedlings. Mutation in *TMS1* causes a drastic retardation of pollen tubes growth and leads to thermosensitive male sterility when the *tms1-1* plants are grown at a higher temperature (30°C) [7]. *TMS1* encodes an Hsp40-homologous protein with a DnaJ domain, a PDI_a_ERdj5_C domain and a P5_C domain [7], which is located in endoplasmic reticulum (ER) [8]. The TMS1 protein exhibits the reductive activity of protein disulfide isomerase (PDI), indicating that it may be involved in protein folding [7]. However, the detailed mechanism of *TMS1* in thermotolerance of pollen tubes and other plant tissues remains unknown.

The DnaJ proteins in the ER have been identified as being conserved from yeast to animals and plants [8]. They could function as the co-chaperones of the molecular chaperones, heat shock protein 70 (Hsp70) [9–12]. In the ER of *Arabidopsis thaliana*, there are five DnaJ proteins (AtERdj3A, AtERdj3B, AtP58^IPK^, AtERdj2A and AtERdj2B) and three Hsp70 proteins (BiP1, BiP2 and BiP3) [8, 13, 14]. Previous studies showed that AtERdj3B plays an important role in plant immunity by interacting with Stromal-Derived Factor-2 (SDF2) and the BiPs. AtERdj3B acts as a bridge between SDF2 and the BiPs to form a protein complex [15]. AtP58^IPK^, a plant ortholog of double-stranded RNA-dependent protein kinase PKR inhibitor, is involved in viral pathogenesis and can partially compensate for the growth defect in *jem1 scj1* yeast mutant [8, 16]. *AtERdj2A* is required for normal pollen germination [8]. AtERdj3A is identical to TMS1. As mentioned above, TMS1 plays an important role in thermotolerance of pollen tubes and seedlings [7]. The BiPs, which are luminal binding proteins and located in the ER, can promote protein folding by acting as molecular chaperones [13, 14, 17]. Under stress conditions, for example heat shock stress, the unfolded proteins and misfolded proteins are accumulated in the ER, resulting in ER stress and activating the signaling pathway of unfolded protein response (UPR) [17–19]. The UPR can upregulate the expression of the transcription factors (such as bZIP28 and bZIP60) and molecular chaperones (such as BiPs and DnaJ proteins) that promote protein folding to alleviate the ER stress [20, 21]. The three *Arabidopsis* BiP proteins (BiP1, BIP2 and BIP3) share a high amino sequence similarity. The expression of the *BiP* genes can be induced by heat shock and other reagents, such as tunicamycin and dithiothreitol (DTT), which can trigger ER stress [13, 14]. Study has shown that they are involved in the fusion of polar nuclei during female gametophyte development in *Arabidopsis* [22]. Recently they are found to be also expressed in pollen and pollen tubes. The *bip1 bip2 bip3* triple mutant is lethal to pollen grains and drastically affects pollen tube competitiveness, indicating that the three *BiP* genes are also required for male gametogenesis and pollen tube growth [10]. All these results imply that *TMS1* has the expression pattern and function similar to those of the *BiP* genes. However, whether TMS1 could interact with the BiP proteins remain unknown.

In this study, the relationship between *TMS1* and the *BiP* genes was explored to understand the molecular mechanisms of *TMS1* in thermotolerance of plants. The expression of *TMS1* was induced not only by heat shock, but also by the ER stress-inducing reagents, DTT and L-azetidine-2-carboxylic acid (AZC), similarly to the three *BiP* genes. The firefly luciferase complementary imaging (LCI), GST pull-down and ATPase enzyme activity assays demonstrate that the DnaJ domain of TMS1 could interact with BiP1 and BiP3 and could stimulate the ATPase enzyme activities of BiP1 and BiP3 in *Arabidopsis*. The results suggest that *TMS1* is involved in termotolerance of plants, possibly by participating in the refolding or degradation of unfolded and misfolded proteins through interaction with the BiP proteins.

## Materials and Methods

### Plant Materials

The *Arabidopsis thaliana* plants used in this study were of Colombia-0 (Col-0) background. The seeds were surface-sterilized and pre-germinated on Murashige and Skoog (MS)-salts agar plates at 22°C under a photoperiod of 16 h light/8 h dark. The SALK T-DNA insertion lines were obtained from the Arabidopsis Biological Resource Center (ABRC, http://abrc.osu.edu/). The double mutants were generated by crosses of the corresponding single mutants.

### RNA Extraction and Real-time PCR Assays

Total RNAs were extracted from seedlings using an RNAprep Pure Plant Kit (Tiangen, Beijing, China) according to the manufacturer’s instruction. The first-strand cDNA was synthesized from 5 μg of total RNAs using the SuperScript III First-Strand Synthesis System (Invitrogen, CA, USA) as described by the manufacturer’s instruction. The Real-time PCR assays were run using 2×*Power* SYBR Green PCR Master Mix (Applied Biosystems, www.appliedbiosystems.com) and the gene-specific primers (S1 Table) on an ABI 7500 Real-time instrument (Applied Biosystems, www.appliedbiosystems.com). The PCR program was set as 95°C for 10 min, followed by 40 cycles of 95°C for 15 s and 60°C for 1 min. The gene expression levels in three biological replicates were normalized to that of *ACTIN2/8* in the same cDNA samples tested. The relative expression levels were calculated with the ΔCt (threshold cycle) method.

### Expression and Purification of TMS1 DnaJ domain, BiP1 and BiP3 Proteins from *E*. *coli*

The coding DNA sequence (CDS) of TMS1 DnaJ Domain (DnaJ) was amplified by polymerase chain reaction (PCR) using the gene-specific primer pairs (S1 Table). The CDS of the mutated TMS1 DnaJ domain (DnaJ-mt) was generated by the overlap PCR method [23] using the gene-specific primer pairs (S1 Table). The amplified CDS fragments were purified and digested with *Eco*RI and *Xho*I, and cloned into the expression vector pGEX4T-1. The resulting constructs were introduced into *E*. *coli* strain BL21 (TransGen Biotech, Beijing, China) to produce recombinant TMS1 DnaJ domain proteins with a GST tag (GST-DnaJ) and the mutated TMS1 DnaJ domain with a GST tag (GST-DnaJ-mt). Purification of the GST-fusion proteins was as described previously [7]. After the GST fusion proteins were purified, the proteins were dialyzed using the dialyzing buffer (20 mM Hepes, pH 6.8, 75 mM KOAC, 250 mM sorbitol, 5 mM MgOAC_2_, 10% glycerol) overnight for further experiments.

The full-length CDSs encoding the BiP1 and BiP3 proteins without the signal peptides were amplified by PCR using the gene-specific primer pairs (S1 Table), respectively. The amplified CDS fragments of *BiP1* and *BiP3* were purified and digested with *Eco*RI and *Xho*I and cloned into the expression vector pET30a (+) to generate the fusion protein-expressing constructs BiP1-His and BiP3-His, respectively. The expression, purification and dialyzation of the fusion proteins with an amino-terminal His^+6^ tag from *E*. *coli* strain BL21 (TransGen Biotech, Beijing, China) were as described previously [24].

### GST Pull-down Assays

The *in vitro* GST Pull-down assays were performed as described by Corsi and Schekman [24] with a small modification. Glutathione Sepharose 4B beads (GE healthcare, 17-0756-01, Sweden) were equilibrated with 1 mL binding buffer (20 mM Hepes, pH 6.8, 100 mM KCl, 5 mM MgCl_2_, 0.1% NP-40, 2% [vol/vol] glycerol, 1 mM DTT, 0.2 mM AEBSF, and 1 mM EDTA) before used. 30 μg GST-DnaJ, GST-DnaJ-TB or GST proteins were added into 50 μL of 50% Glutathione Sepharose 4B bead suspension in the binding buffer. Then the volume was increased to 1 mL with the binding buffer. The reaction tubes were rotated at 4°C for 1 h. The unbound proteins were removed by centrifugation at 3000 *g* for 2 min at 4°C followed by two washes with 500 μL binding buffer. The purified BiP1 (30 μg) or BiP3 (30 μg) and ATP (1 mM) were then added in a final volume of 1 mL, and then were rotated at 4°C for 2 h. The unbound proteins were removed by a series of four washes with 1 mL binding buffer mentioned above. The proteins binding to the beads were solubilized in Laemmli sample buffer (BioRad Labs, Hercules, CA) by boiling, and separated by SDS-PAGE. The in-put GST, GST-DnaJ and GST-DnaJ-mt proteins were examined by Western blot using the anti-GST antibody (Sigma, A5838, USA). The BiP1-His and BiP3-His proteins were detected by Western blot using the anti-His antibody (Sigma, A5588, USA).

### ATPase Enzyme Activity Assays

The ATPase enzyme activity assay kit from Innova Biosciences (Innova Biosciences, Cambridge, UK) was used to perform *in vitro* ATPase enzyme activity assays. According to the manufacturer’s instruction, in each reaction, 10 μg BiP1-His or BiP3-His proteins were mixed with an different amount of the GST-DnaJ or GST-DnaJ-mt or naked GST proteins in 100 mL assay buffer (pH 6.8) supplied with 5 mM MgCl_2_ and 1 mM ATP, respectively. The concentration of the GST-DnaJ or GST-DnaJ-mt or naked GST proteins was increased in a gradient, while the concentration of BiP1-His or BiP3-His proteins was kept constant. The reaction mixtures were incubated at 25°C for 45 min, which was determined to be in the linear range of the reactions. ATP hydrolysis (%) was measured by OD_630_ values using a plate reader (Bio-Rad labs, Hercules, CA) as described by the manufacturer’s instruction. The ATPase enzyme activities were calculated using the standard curve generated by the concentration gradients of inorganic phosphate (Pi).

### Luciferase Complementary Imaging Assays

The full-length CDSs of BiP1, BiP3 and TMS1 (without the signal peptides) and CDS of TMS1 DnaJ domain were amplified by PCR using gene-specific primer pairs (S1 Table). The amplified *BiP1* and *BiP3* DNA fragments were purified and digested with *Kpn*I and *Sal*I, and inserted into 35S::Nluc vector. The amplified DNA fragments were purified, digested with *Kpn*I and *Bam*HI, and inserted into 35S::Cluc vector. *Agrobacteria*-mediated transient expression, CCD imaging and LUC activity measurement were carried out as described by Chen et al. [25].

## Results

### The Expression of *TMS1* was Induced by Heat Shock, DTT and AZC

**Fig 1:**
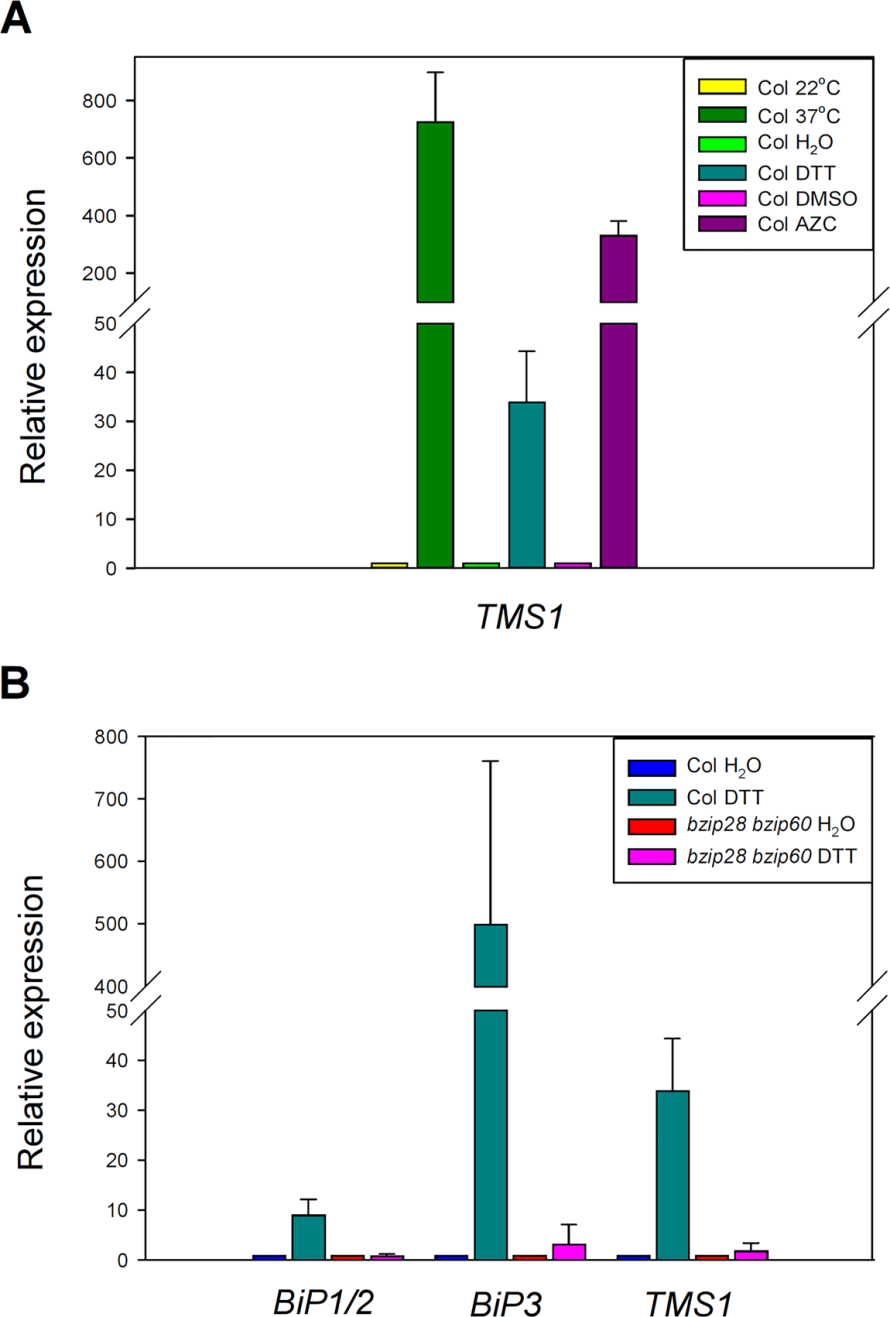
The expression of *TMS1* was induced by heat shock, DTT and AZC, and reduced in *bzip28 bzip60* double mutant. (A) A real-time PCR Assay that showed the induction of *TMS1* expression by heat shock, DTT and AZC treatment. (B) A real-time PCR Assay that showed reduction in expression levels of *BiP1/BiP2*, *BiP3* and *TMS1* in *bzip28 bzip60* double mutant. Col, wild type Col seedlings; *bzip28 bzip60*, *bzip28 bzip60* double mutant seedlings; H_2_O, distilled water used as the negative controls in the assays. Expression levels were normalized to that of *ACTIN2*. The error bars present the SD of the mean of three biological replicates.

Previous studies have demonstrated that the expression of *TMS1* can be induced by heat shock and tunicamycin [7, 8]. To further investigate the involvement of *TMS1* in unfolded protein response in endoplasmic reticulum and in cytoplasm, we performed the DTT and AZC induction assays. DTT is a reductant agent that can prevent formation of disulfide bonds. Treatment of DTT can induce unfolded protein response (UPR) in endoplasmic reticulum [26]. AZC is a proline analog. Treatment of AZC can lead to unfolded protein response in endoplasmic reticulum and in cytoplasm (CPR) [27]. As shown in Fig 1A, when the 10-day-old wild-type seedlings were treated with 37°C for 1 h, 5 mM DTT for 2 h and 10 mM AZC for 3 h, the *TMS1* mRNA levels were increased 724-, 34- and 330-folds, respectively, compared to those in the responding control seedlings grown at 22°C or treated with H_2_O or DMSO. The results indicated that the expression of *TMS1* was induced not only by heat shock, but also by DTT and AZC treatments, indicating that *TMS1* is related to UPR and CPR.

### Expression of *BiP1/BiP2*, *BiP3* and *TMS1* was Reduced in the *bzip28 bzip60* double mutant

The transcription factors bZIP28 and bZIP60 have been identified as the important UPR regulators for mitigating the ER stress in *Arabidopsis thaliana* [26, 28–32]. Previous study showed that the expression of *BiP3*, a marker gene for UPR, was reduced in the *bzip28 bzip60* double mutant [33]. Among the *Arabidopsis BiP1*, *BiP2* and *BiP3*, *BiP1* and *BiP2* are located closely to each other. Furthermore, their sequence similarity reaches to 99% [13, 14]. Therefore, only *BiP1* and *BiP3* were selected for the following assays. We measured and compared the expression of *TMS1*, *BiP1* and *BiP3* in the *bzip28 bzip60* double mutant.

The *bzip28 bzip60* double mutant used in this assay was generated using the SALK T-DNA insertion lines of *bZIP28* (SALK_132285C) and *bZIP60* (SALK_050203C) from ABRC (S1 Fig). The 10-day-old wild type and *bzip28 bzip60* double mutant seedlings were treated with 5 mM DTT for 2 h. Meanwhile, H_2_O was used as a negative control. As shown in Fig 1B, in wild type seedlings, the expression of the *BiP1/2*, *BiP3* and *TMS1* was increased 9-, 500- and 34-folds when treated with 5 mM DTT, respectively, compared to those in the control seedlings, whereas in *bzip28 bzip60* seedlings, the expression of the *BiP1/2*, *BiP3* and *TMS1* was increased 1-, 3- and 2-folds when treated with 5 mM DTT, respectively. Therefore, the expression of the *BiP1/2*, *BiP3* and *TMS1* was significantly reduced in the *bzip28 bzip60* double mutant, suggesting that TMS1 may participate in the bZIP28/bZIP60-related signal pathway.

### TMS1 and its DnaJ Domain could Interact with BiP1 and BiP3 in LCI Assay System

**Fig 2:**
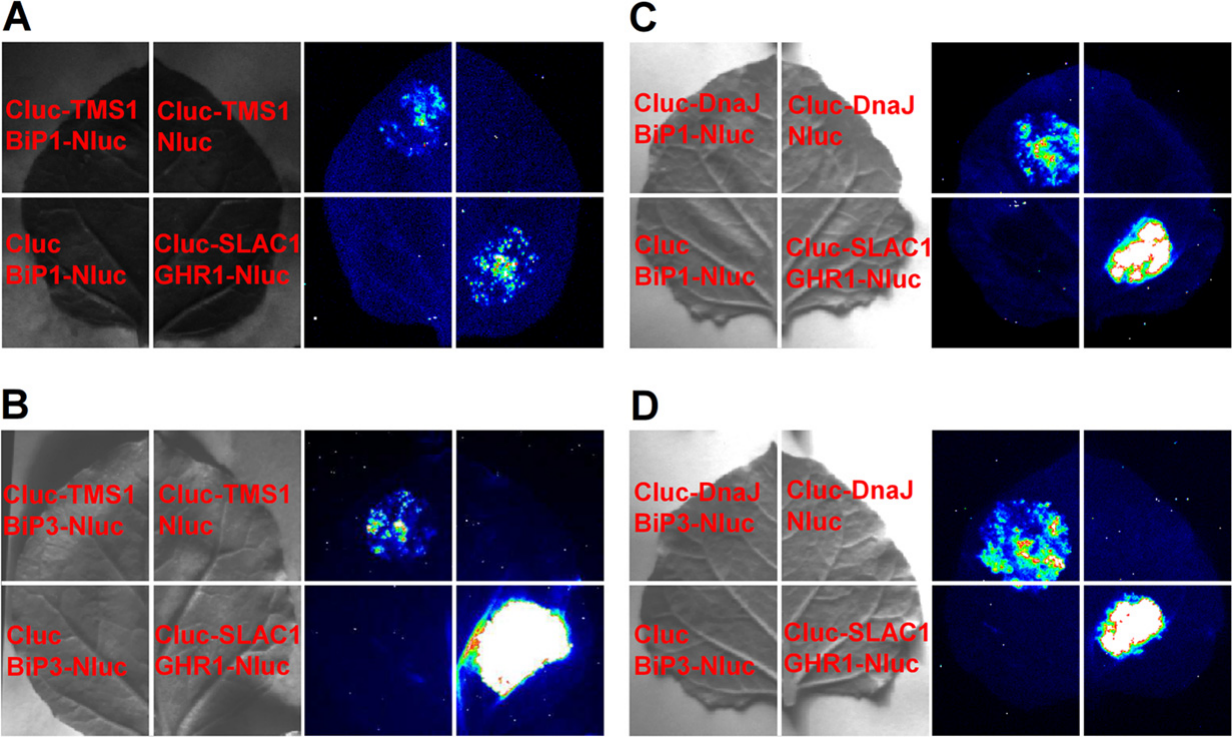
Interaction of TMS1 and its DnaJ domain with BiP1 and BiP3 in *N*. *benthamiana* leaves. (A) The LCI assay showed that TMS1 interacted with BIP1. (B) The LCI assay showed that TMS1 interacted with BIP3. (C) The LCI assay showed that the DnaJ domain of TMS1 interacted with BIP1. (D) The LCI assay showed that the DnaJ domain of TMS1 interacted with BIP3. The LUC signal images were collected 3 days after infiltrated with *Agrobacterium* bearing the tested constructs using a low-light imaging system after adding luciferin. Yellow, strong interaction; red, intermediate interaction; green, weak interaction.

Firefly Luciferase Complementary imaging (LCI) Assay [25] was performed to verify if the TMS1 acts as a DnaJ-like partner and interacts with the BiPs. The CLuc-TMS1 and Cluc-DnaJ were paired with the BiP1-Nluc and BiP3-Nluc, respectively and coexpressed in *N*. *benthamiana* leaves using *Agrobacteria*-mediated transient expression method [25]. CLuc-TMS1/Nluc, CLuc-DNAJ/Nluc, Cluc/BiP1-NLuc and Cluc/BiP3-Nluc pairs were used as the negative controls. Cluc-SLAC1/GHR1-Nluc pair was used as a positive control [34]. As shown in Fig 2A, coexpression of the positive control pair Cluc-SLAC1/GHR1-Nluc and the test pair CLuc-TMS1/BiP1-Nluc exhibited strong LUC signals as revealed using a low-light imaging system after adding luciferin, the substrate for firefly LUC. In contrast, the negative control pairs CLuc-TMS1/Nluc and Cluc/BiP1-NLuc showed only the background level LUC activity. This result indicated that TMS1 could interact with BiP1 in LCI assay system. Similarly, TMS1 also could interact with BiP3 as demonstrated by the same assay system (Fig 2B). Furthermore, the DnaJ domain of TMS1 also could interact with BiP1 and BiP3 in the same system, respectively (Fig 2C and 2D).

### The DnaJ domain of TMS1 could Bind to BiP1 and BiP3 *in vitro*

**Fig 3:**
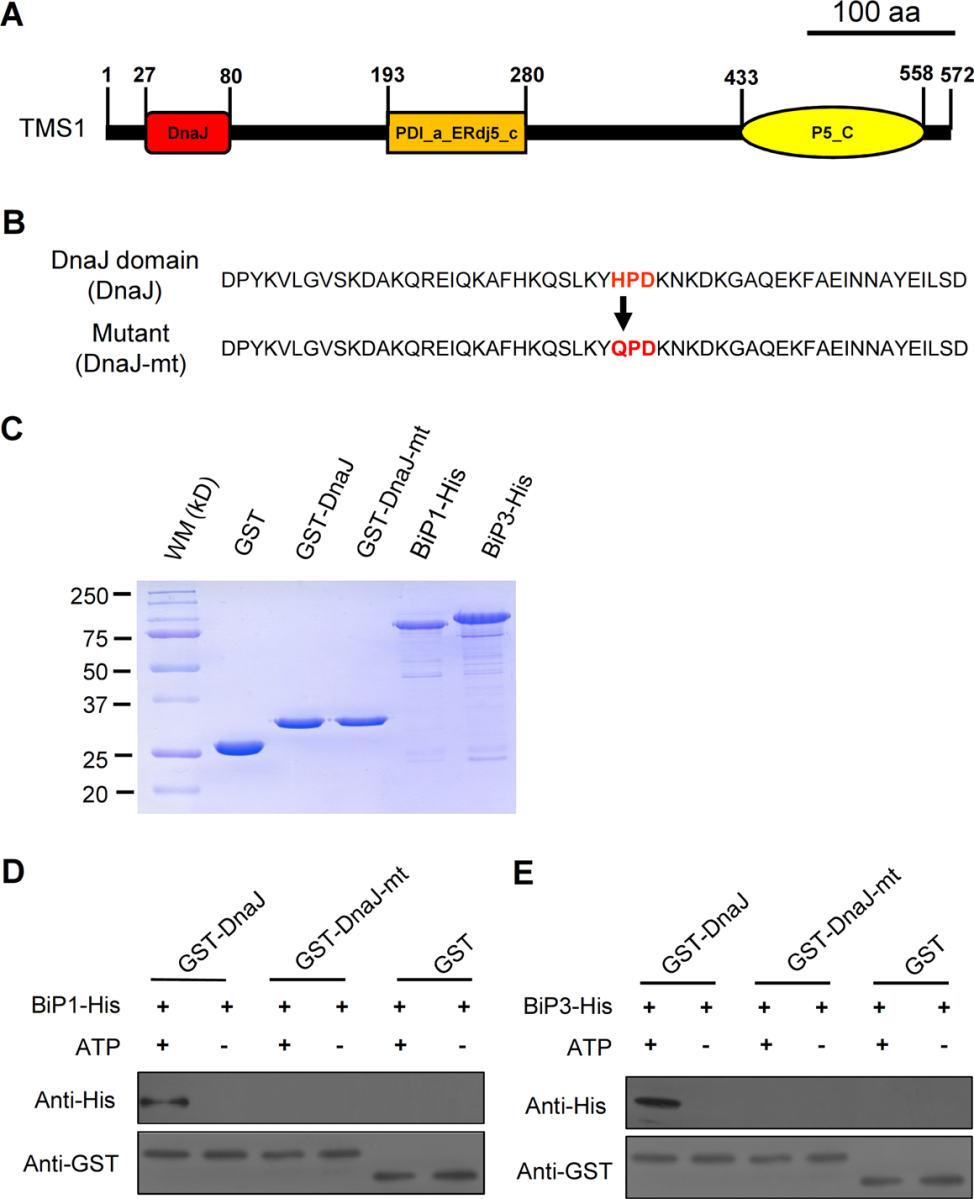
The DnaJ domain of TMS1 could bind to BiP1 and BiP3 *in vitro*. (A) A schematic structure of the TMS1 protein, indicating the position of the DnaJ domain in the TMS1. (B) The amino acid sequence of the TMS1 DnaJ Domain and the loss-of-function mutant DnaJ Domain. The red characters indicate the position of the HPD motif and the mutation site in the mutant protein. (C) The quality assay for the purified proteins. (D) The *in vitro* GST pull-down demonstrated that DnaJ domain of TMS1 could bind to BiP1 when supplied with ATPs. (E) The *in vitro* GST pull-down demonstrated that DnaJ domain of TMS1 could bind to BiP3 when supplied with ATPs. 20-fold dilutions of the protein samples were used for the anti-GST assays in (D) and (E). WM, molecular weight marker; kD, kilodaltons.

To further verify the binding of TMS1 DnaJ domain to the BiPs, we performed the *in vitro* GST pull-down experiments. The CDS of the TMS1 DnaJ domain containing 54 amino acid residues (the 27–80 aa in TMS1) (Fig 3A) was fused with the *GST* CDS to construct the expression vector GST-DnaJ. Studies showed that a conserved three-amino acid motif HPD in the DnaJ domains is essential for the interaction of DnaJ proteins with BiPs [24, 35, 36]. Therefore, a parallel mutant fusion protein expression vector GST-DnaJ-mt was constructed for assessing the specificity of the DnaJ domain interaction with the BiPs, in which the HPD motif was mutated to QPD (Fig 3B). The GST-expressing vector GST was constructed to produce the GST protein, which was used as a negative control. The CDSs of BiP1 and BiP3 without signal peptides were cloned into pET30a (+) vector to construct the BiP-expressing vectors, BiP1-His and BiP3-His, respectively. All the constructs were introduced into the *E*. *coli* strain BL21 (DE3) to produce and purify the proteins for pull-down and ATPase enzyme activity assays (see Materials and Methods). The quality of the purified proteins was measured by SDS-PAGE fraction and Coomassie brilliant blue (CBB) staining (Fig 3C). As shown in Fig 3D and 3E, in presence of 1 mM ATPs, the GST-DnaJ fusion protein could bind to the BiP1 and BiP3 proteins, respectively. In contrast, the GST-DnaJ could not bind to the BiP1 or BiP3 in the absence of ATP (Fig 3D and 3E). Furthermore, the GST-DnaJ-mt and GST, the negative control proteins, also did not bind to the BiP1 and BiP3, either in the presence or absence of ATPs (Fig 3D and 3E). These results indicated that the TMS1 DnaJ domain could bind to the BiP1 and BiP3 in an ATP-dependent manner.

### The TMS1 DnaJ domain could Stimulate the ATPase Enzyme Activities of BiP1 and BiP3

**Fig 4:**
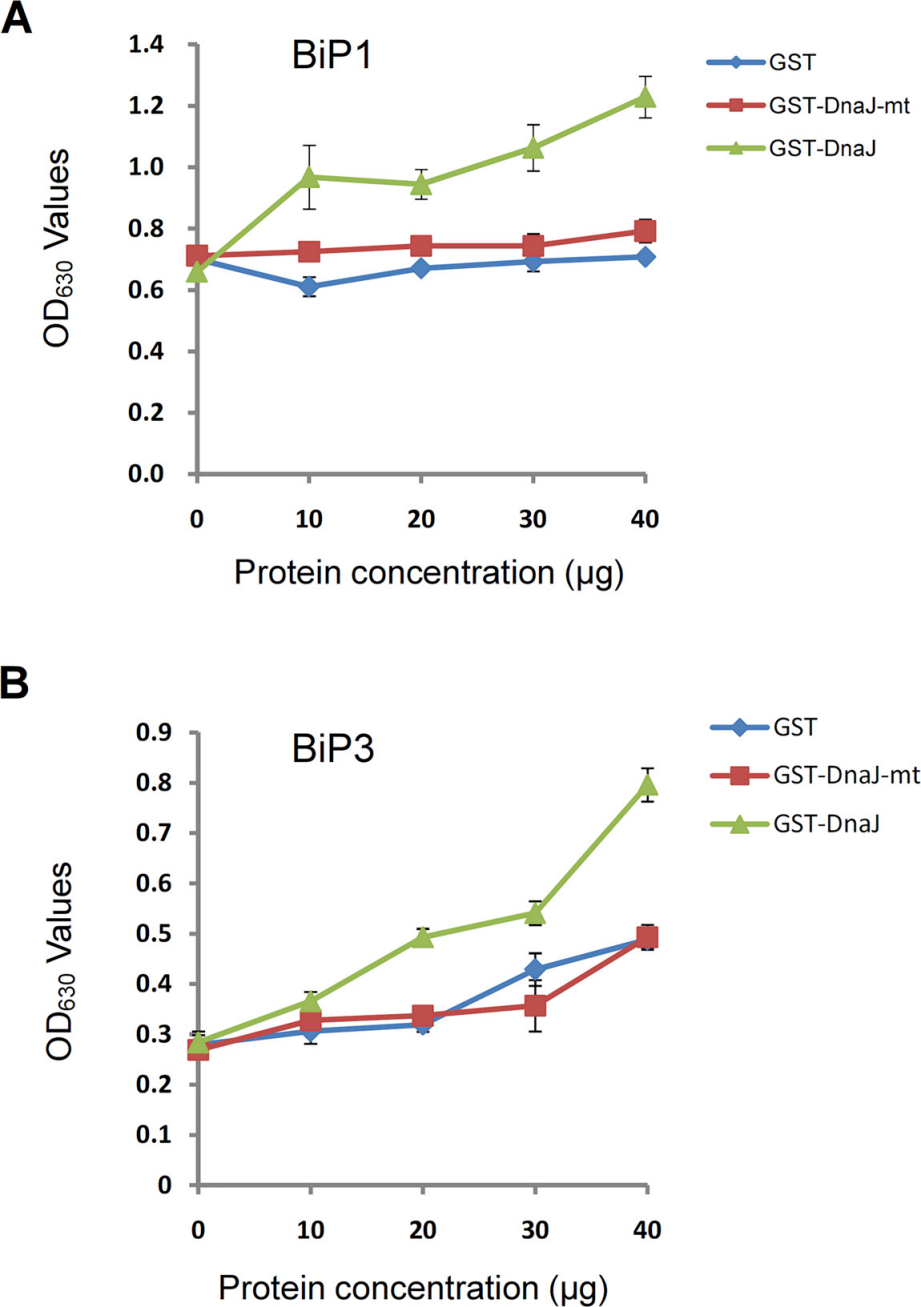
The DnaJ domain of TMS1 could stimulate the ATPase enzyme activities of BiP1 and BiP3. (A) The DnaJ domain of TMS1 stimulated the ATPase enzyme activity of BiP1. (B) The DnaJ domain of TMS1 stimulated the ATPase enzyme activity of BiP3.

The ATPase enzyme activity assays were performed to analyze the correlation of the ATPase enzyme activities of the BiP proteins with the concentration of the GST-DnaJ. The ATPase enzyme activities of the BiP proteins were measured by monitoring the final phosphate concentration in the reaction systems using the plate reader after incubated for 45 minutes (see Materials and Methods). As shown in Fig 4A and 4B, in a constant concentration of the BiP proteins, the ATPase enzyme activities of the BiP proteins were enhanced by increasing the concentration of GST-DnaJ. In contrast, increasing of GST and GST-DnaJ-mt concentration did not stimulate the ATPase enzyme activities of the BiP proteins. Therefore, the TMS1 DnaJ domain could stimulate the ATPase enzyme activities of BiP1 and BiP3.

### The Expression Pattern of *TMS1* was Different from that of *ERdj3B*

**Fig 5:**
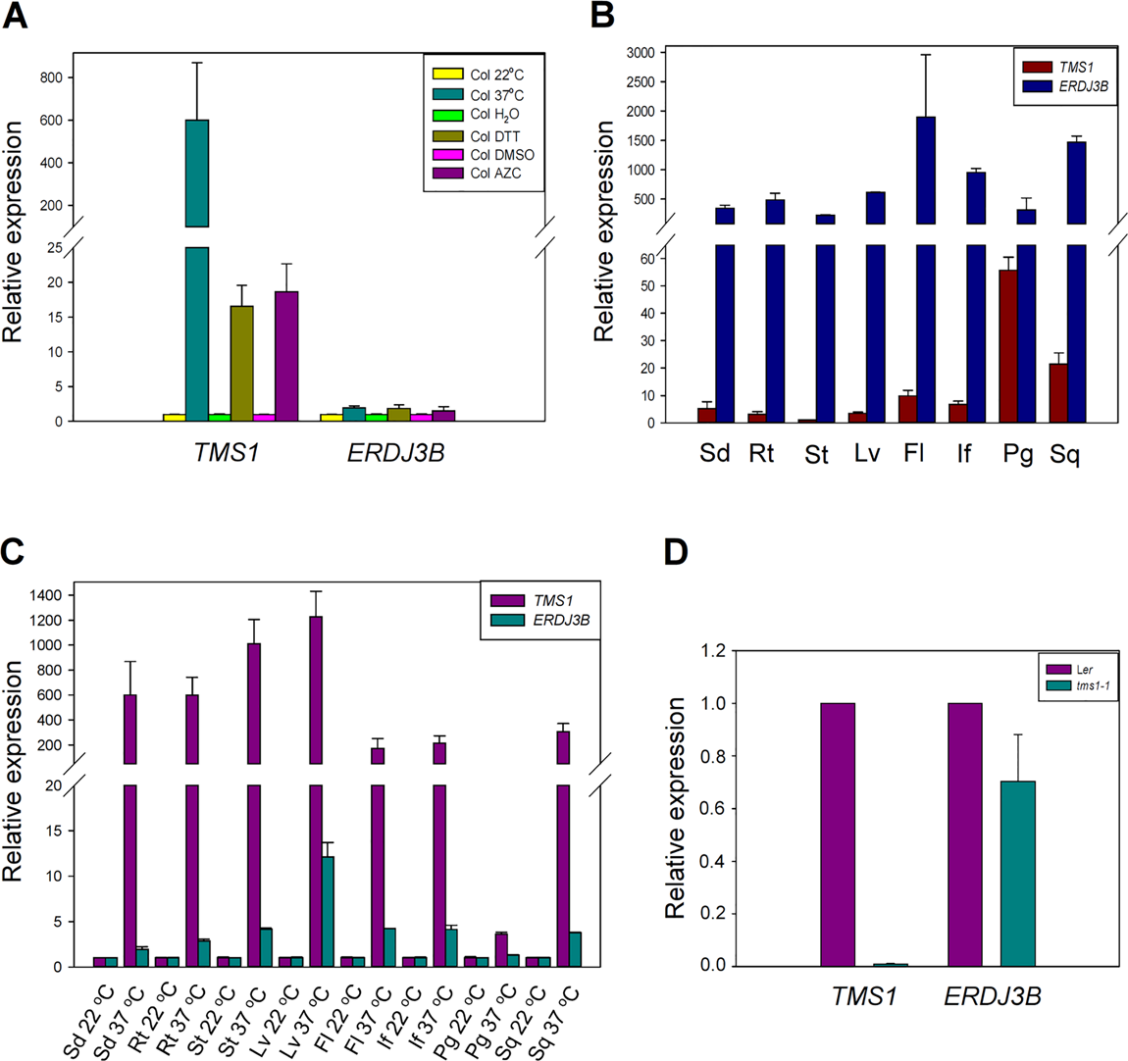
The expression pattern of *TMS1* was different from that of *ERdj3B*. (A) A comparison in expression levels of *TMS1* and *ERdj3B* in seedlings after heat shock, DTT treatment and AZC treatment. (B) A comparison in expression levels of *TMS1* and *ERdj3B* in different plant tissues. (C) A comparison in expression levels of *TMS1* and *ERdj3B* in different plant tissues after 37°C heat shock treatment. (D) The expression of *ERdj3B* was slightly downregulated in *tms1-1*. Fl, flowers; If, inflorescences; Lv, leaves; Rt, roots; Pg, pollen grains; Sd, seedlings; Sq, siliques; St, stems.

Both TMS1 (ERdj3A) and ERdj3B are orthologs of yeast Scj1p [8]. Orthologs of ERdj3B are found in human, mouse, fruit fly, nematode and plant genomes. However, the orthologs of TMS1 are only found in plant genomes [8]. Study has shown that ERdj3B, SDF2 and BiPs can form a complex to play roles in plant immunity, but TMS1 does not interact with SDF2 in Y_2_H system [15]. To further study the relationship between *TMS1* and *ERdj3B*, their expression patterns were compared. The expression of both *TMS1* and *ERdj3B* could be induced by heat shock, DTT, and AZC in seedlings, but the expression levels of *ERdj3B* were much lower than those of *TMS1* (Fig 5A). Both *TMS1* and *ERdj3B* were expressed in seedlings, roots, stems, leaves, flowers, inflorescences, pollen grains and siliques from wild type plants. However, the expression levels of *TMS1* were different in the various tissues, with the highest level in pollen grains and the lowest level in stems (Fig 5B), showing a pattern different from that of *ERdj3B*. Furthermore, when treated various wild type plant tissues by 37°C heat shock for 1 hour, the expression of *TMS1* in various tissues was increased more efficiently than that of *ERdj3B* (Fig 5C). In addition, the expression of *ERdj3B* was slightly downregulated in *tms1-1* (Fig 5D). Taken together, *TMS1* and *ERdj3B* had different expression patterns.

## Discussion

Study showed that approximately one-third of total proteins are synthesized in the ER as secretory and membrane proteins [37]. The proper folding of the newly synthesized polypeptides in ER is important for transport and function of these secretory and membrane proteins. Moreover, ER also plays an important role in maintaining the balance between protein folding demands and folding capacity. When the balance is disturbed by environmental conditions, for an example heat shock, the unfolded and misfolded proteins accumulate in the ER, causing ER stress. For such an ER stress, the cells have evolved a precise ER quality control system (ERQC) [38]. Accumulation of unfolded and misfolded proteins in the ER can activate the unfolded protein response (UPR) [17–19], which upregulates the production of the factors that promote protein folding or remove unfolded and misfolded proteins from ER through ER-associated degradation (ERAD) [39].

The molecular chaperone proteins, such as BiPs and DnaJ proteins, involve the processing of the accumulated unfolded and misfolded proteins in the ER. Such the molecular chaperone proteins increase to assist in the unfolded and misfolded protein refolding or depredating via ERAD when the cells response to ER stress. BiPs are ER-resident Hsp70 cognates that assist in protein folding, while DnaJ proteins are the co-chaperones of the BiPs. The DnaJ proteins can interact with the BiPs and stimulate their ATPase enzyme activities to assist in the refolding of unfolded and misfolded proteins in the ER [39, 40]. Therefore, both the BiPs and DnaJ proteins play important roles in response to ER stress caused by the environmental stresses.

Previous study showed that TMS1 is important for the thermotolerance of pollen tubes and seedlings. It is an Hsp40-homologous protein with a DnaJ domain and an a_ERdj5_C domain found in protein disulfide isomerases (PDI). The a_ERdj5_C domain of TMS1 has the reductive activity like PDI, indicating that TMS1 may function as a disulfide isomerase involved in the protein processing [7]. In this study, we demonstrated that the DnaJ domain of TMS1 can interact with the molecular chaperones BiP1 and BiP3 and stimulates the ATPase enzyme activities of the BiPs, indicating that TMS1 may also function as a cochaperone of the BiPs and is involved in ER stress. Therefore, TMS1 may be involved in protein process and plays important roles in the tolerance of the environmental stresses through interaction with the BiPs.

The expression of the *BiPs* was under the control of bZIP28 and bZIP60 as demonstrated by that the *bzip28 bzip60* mutant drastically reduced the expression levels of the *BiPs* [23, 28, 30]. The bZIP28 and bZIP60 are bZIP family transcription factors that were involved in unfolded protein response in the ER. When the unfolded or misfolded proteins are accumulated in the ER, the *bZIP28* and *bZIP60* promote expression of the BiP and DnaJ proteins. Thus, the cells can response to ER stress better and quickly [23, 25–30]. Our previous study showed that expression of *TMS1* can be induced by heat shock treatment [7]. In this study, our results showed that DTT and AZC treatments also could induce the expression of *TMS1*. It has been reported that DTT and AZC treatments could result in ER stress response [23, 24]. Furthermore, our results demonstrated that the expression level of *TMS1* also was reduced drastically in the *bzip28 bzip60* double mutant, similar to the *BiP*s. Taken together, like the *BiP*s, *TMS1* may be involved in ER stress at downstream of the transcription factors genes *bZIP28* and *bZIP60*.

As mentioned above, both TMS1 and ERdj3B are orthologs of yeast Scj1p. Orthologs of ERdj3B are found in human, mouse, fruit fly, nematode and plant genomes. However, the orthologs of TMS1 are only found in plant genomes [8]. Study has shown that ERdj3B, SDF2 and BiPs can form a complex to play roles in plant immunity, but TMS1 does not interact with SDF2 in Y_2_H system [15]. In this study, we further demonstrated that they have different expression patterns. Firstly, although their expression could be induced by heat shock, DTT, and AZC in seedlings, the expression level of *ERdj3B* was much lower than that of *TMS1* (Fig 5A). Secondly, although both of them were expressed in all the same tissues including seedlings, roots, stems, leaves, flowers, inflorescences, pollen grains and siliques, the expression levels of *ERdj3B* persisted at a similar level in all the tissues tested, while the expression levels of *TMS1* were different in the various tissues, with the highest level in pollen grains and the lowest level in stems (Fig 5B). Thirdly, the expression of *TMS1* in various tissues was more sensitive to heat shock treatment (37°C heat shock for 1 hour), than that of *ERdj3B* (Fig 5C). Furthermore, we found that the expression of *ERdj3B* was slightly downregulated in *tms1-1* (Fig 5D). All these results indicated that *TMS1* may be functionally different from *ERdj3B*. They may participate in different biological processes.

## Supporting Information

S1 Fig Molecular characterization of the *bzip28 bzip60* double mutant. (A) The schematic structures of the *bZIP28* and *bZIP60* genes, which show the T-DNA insertion sites in the *bzip28* and *bzip60* mutants. Black boxes indicate exons, while the lines between the black boxes indicate the introns. The arrowheads indicate the positions of the primers used for genotyping in (B). (B) Confirmation of the T-DNA insertion sites in *bzip28 bzip60* double mutant by PCR. LBa1, P1, P2, P3 and P4 are the primers used in the PCR assays (S1 Table). (C) The Real-time PCR assay for the impact on the *bZIP28* and *bZIP60* transcription in the homozygous *bzip28 bzip60* double mutant seedlings. The *ACTIN2* (*At3g18780*) gene was used as an internal normalization control.
(TIF)

S1 Table The primers used in this study. (DOC)
