# Supplementary figures and images for: The THERMOSENSITIVE MALE STERILE 1 Interacts with the BiPs via DnaJ Domain and Stimulates Their ATPase Enzyme Activities in Arabidopsis
Source: PLoS One. 2015 Jul 17;10(7):e0132500. doi: 10.1371/journal.pone.0132500 (PMC4505944; doi:10.1371/journal.pone.0132500)

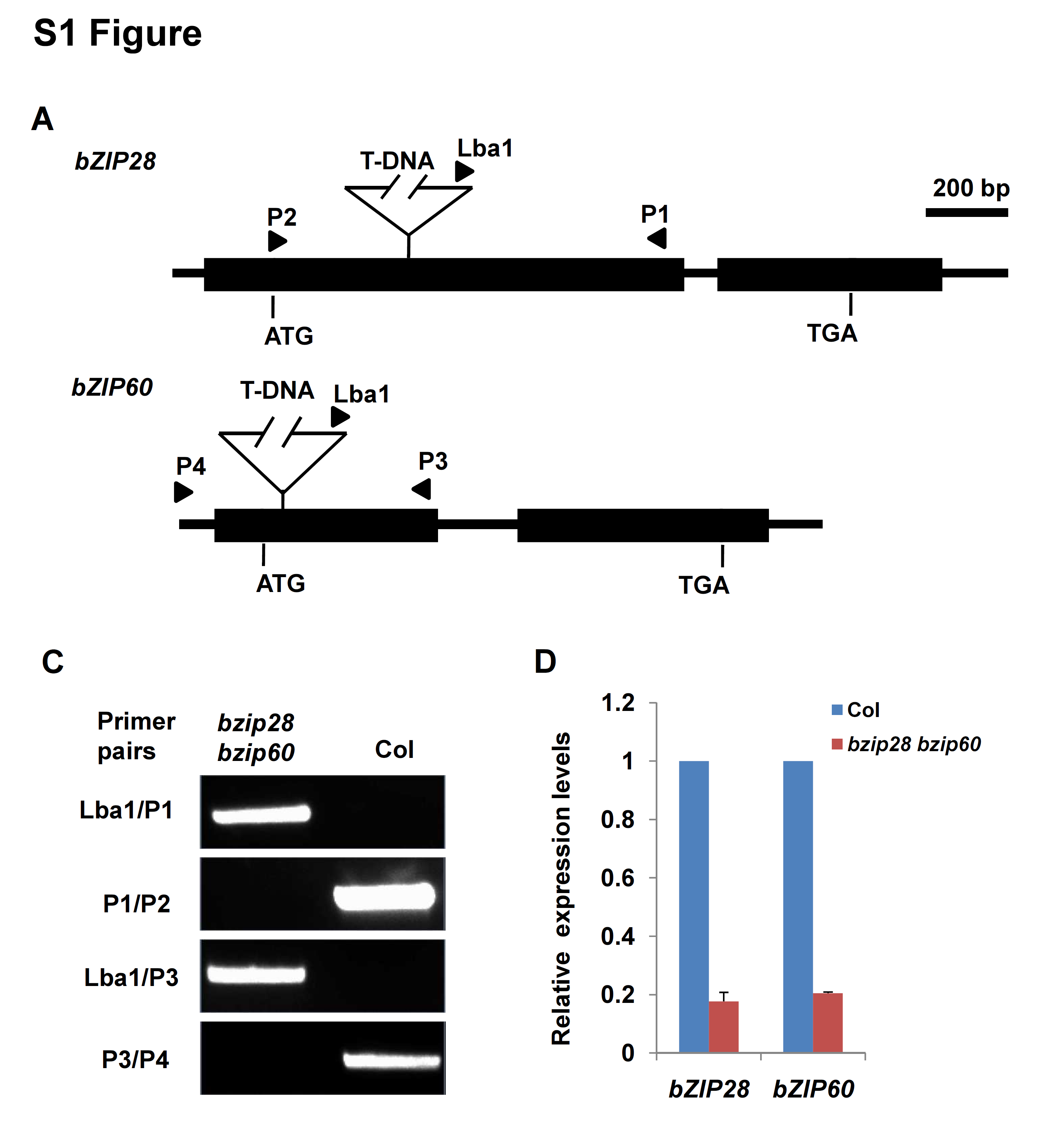

Supplement: S1 Fig — (A) The schematic structures of the bZIP28 and bZIP60 genes, which show the T-DNA insertion sites in the bzip28 and bzip60 mutants. Black boxes indicate exons, while the lines between the black boxes indicate the introns. The arrowheads indicate the positions of the primers used for genotyping in (B). (B) Confirmation of the T-DNA insertion sites in bzip28 bzip60 double mutant by PCR. LBa1, P1, P2, P3 and P4 are the primers used in the PCR assays (S1 Table). (C) The Real-time PCR assay for the impact on the bZIP28 and bZIP60 transcription in the homozygous bzip28 bzip60 double mutant seedlings. The ACTIN2 (At3g18780) gene was used as an internal normalization control. (TIF) [file pone.0132500.s001.tif]
