# Supplementary material for: The THERMOSENSITIVE MALE STERILE 1 Interacts with the BiPs via DnaJ Domain and Stimulates Their ATPase Enzyme Activities in Arabidopsis
Source: PLoS One. 2015 Jul 17;10(7):e0132500. doi: 10.1371/journal.pone.0132500 (PMC4505944; doi:10.1371/journal.pone.0132500)
Supplement: S1 Table — (DOC) [file pone.0132500.s002.doc]

**Supplemental Tables**

**S1 Table. The primers used in this study**

| Used for | Genes | Names or Directions | Sequences (5’- 3’) |
| --- | --- | --- | --- |
| T-DNA insertion assays | T-DNA | Lba1 | TGGTTCACGTAGTGGGCCATCG |
|  | *bZIP28* | P1 | CCTTATGTTCATCGTAATAC |
|  |  | P2 | ATGACGGAATCAACATCCG |
|  | *bZIP60* | P3 | TAGCCACAGCATCATCGTC |
|  |  | P4 | atgttgacggtgacaaagag |
| Real-time PCR assays | *ACTIN2* | Forward | GGTAACATTGTGCTCAGTGGTGG |
|  |  | Reverse | AACGACCTTAATCTTCATGCTGC |
|  | *TMS1* | Forward | AAAAGCTTCTGCCAGGAACA |
|  |  | Reverse | TTGCCGGAAACTTGTCTACC |
|  | *bZIP60* | Forward | TCCGGCGGAGGATTTTCTTCA |
|  |  | Reverse | GCCAAATCAACGGAGCCAGA |
|  | *bZIP28* | Forward | TGAGGAGATCGGTAACAATCG |
|  |  | Reverse | CAGAAGATTTCTGGTGATTGC |
|  | *BiP1* | Forward | TAAGAAGGGTGGCGAGAAGA |
|  |  | Reverse | CACCCTGTGGTCAAAGTCCT |
|  | *BiP3* | Forward | GGAGAAGCTTGCGAAGAAGA |
|  |  | \|  \| \| --- \|   Reverse | ATAACCGGGTCACAAACCAA |
|  | *TIN1* | Forward | TGATGTGGATGCTGGTGTTT |
|  |  | Reverse | TTGATCAGTCGAAGCACGTC |
|  | *ERDJ3B* | Forward | GAAGGGATGGCAACGATCTA |
|  |  | Reverse | GAGGTTGCCTTTCTTTGTGC |

**Table S1.** The primers used in this study (continued)

| Used for | Genes | Names or Directions | Sequences (5’- 3’) |
| --- | --- | --- | --- |
| LUC assays | *BiP1* | Forward | GGGTACCGCAATAGAAGAGGCTACGAA |
|  |  | Reverse | GTCGACGAGCTCATCGTGAGACTCATC |
|  | *BiP3* | Forward | GGGTACCGAAGGCGAAGAGCAGAAACT |
|  |  | Reverse | GTCGACTAACTCATCGTGATCATCT |
|  | DnaJ Domain of *TMS1* | Forward | GGGTACCGATCCTTACAAGGTTCTTGG |
|  |  | Reverse | CGGATCCATCAGATAAGATCTCATACG |
|  | *TMS1* | Forward | GGGTACCGATCCTTACAAGGTTCTTGG |
|  |  | Reverse | CGGATCCTCATTTGATCTGGGGTTTCT |
|  | *TIN1* | Forward | GGGTACCTTAATTTCGTCTCCTGATGC |
|  |  | Reverse | GCGAGCTCTTAATTTCGTCTCCTGATGC |
| Production of recombinant proteins | *BiP1* | Forward | CGAATTCGCAATAGAAGAGGCTACGAA |
|  |  | Reverse | CCGCTCGAGCTAGAGCTCATCGTGAGACT |
|  | *BiP3* | Forward | CGAATTCGAAGGCGAAGAGCAGAAACT |
|  |  | Reverse | CCTCGAGGTAACTCATCGTGATCATCTC |
|  | DnaJ Domain of *TMS1* | Forward | CCGGAATTCGATCCTTACAAGGTTCTTGGA |
|  |  | Reverse | CCGCTCGAGTCAATGGTGGTGATGATGGTG  ATCAGATAAGATCTCATACG |
|  | *DnaJ-TB* mutant | Forward | CTCTGAAATATCAGCCAGATAAAAACAAAG |
|  |  | Reverse | CTTTGTTTTTATCTGGCTGATATTTCAGAG |
|  |  |  |  |
